# Applying Microfluidics for the Production of the Cationic Liposome-Based Vaccine Adjuvant CAF09b

**DOI:** 10.3390/pharmaceutics12121237

**Published:** 2020-12-19

**Authors:** Signe Tandrup Schmidt, Dennis Christensen, Yvonne Perrie

**Affiliations:** 1 Strathclyde Institute of Pharmacy and Biomedical Sciences, University of Strathclyde, Glasgow G4 0RE, UK; sxs@ssi.dk; 2 Department of Infectious Disease Immunology, Center for Vaccine Research, Statens Serum Institut, Artillerivej 5, 2300 Copenhagen S, Denmark

**Keywords:** subunit vaccine, liposome, microfluidics

## Abstract

Subunit vaccines require particulate adjuvants to induce the desired immune responses. Pre-clinical manufacturing methods of adjuvants are often batch dependent, which complicates scale-up for large-scale good manufacturing practice (GMP) production. The cationic liposomal adjuvant CAF09b, composed of dioctadecyldimethylammonium bromide (DDA), monomycoloyl glycerol analogue 1 (MMG) and polyinosinic:polycytidylic acid [poly(I:C)], is currently being clinically evaluated in therapeutic cancer vaccines. Microfluidics is a promising new method for large-scale manufacturing of particle-based medicals, which is scalable from laboratory to GMP production, and a protocol for production of CAF09b by this method was therefore validated. The influence of the manufacture parameters [Ethanol] (20–40% *v/v*), [Lipid] (DDA and MMG, 6–12 mg/mL) and dimethyl sulfoxide [DMSO] (0–10% *v/v*) on the resulting particle size, colloidal stability and adsorption of poly(I:C) was evaluated in a design-of-experiments study. [Ethanol] and [DMSO] affected the resulting particle sizes, while [Lipid] and [DMSO] affected the colloidal stability. In all samples, poly(I:C) was encapsulated within the liposomes. At [Ethanol] 30% *v/v*, most formulations were stable at 21 days of manufacture with particle sizes <100 nm. An in vivo comparison in mice of the immunogenicity to the cervical cancer peptide antigen HPV-16 E7 adjuvanted with CAF09b prepared by lipid film rehydration or microfluidics showed no difference between the formulations, indicating adjuvant activity is intact. Thus, it is possible to prepare suitable formulations of CAF09b by microfluidics.

## 1. Introduction

The liposomal vaccine adjuvant CAF09b (Statens Serum Institut, Copenhagen, Denmark) is capable of inducing robust cytotoxic T-lymphocyte (CTL) responses [1], and is currently in first-in-man phase I clinical trials in a therapeutic vaccine combined with the peptide-based tumor associated antigen BCL-Xl against prostate cancer (NCT03412786) and in a neoepitope-based peptide cancer vaccine (NCT03715985). It is composed of the lipid surfactants dioctadecyldimethylammonium bromide (DDA) and monomycoloyl glycerol analogue 1 (MMG) combined with the TLR3 agonist polyinosinic:polycytidylic acid [poly(I:C)] electrostatically adsorbed to the cationic DDA headgroups [1,2].

CAF09b is prepared using bulk lipid hydration with high shear mixing (HSM) to produce liposomes at nanoscale size [1,3]. Similar to extrusion, sonication or vortexing, which are also methods often used to produce liposomes, the mechanism of action for high shear mixing is to break apart preformed large vesicles to form small-sized liposomes by using shear forces [4,5]. With this method, a lipid film is formed by evaporating organic solvent from dissolved lipids, forming sheets of stacked lipid bilayers deposited on the surface of the preparation vial [5]. The dry lipid film is rehydrated in a suitable aqueous buffer above the transition temperature of the lipids in the formulation with simultaneous application of shear forces to dislodge the lipid bilayers. Continued application of shear forces further reduces the size of the formed liposomes. The process is characterized as a top-down process, as larger structures of lipid bilayers are broken apart to form smaller liposomes [4]. Reduction of liposome particle sizes is regulated by varying the energy input, i.e., the applied shear force in high shear mixing.

In contrast, manufacture of liposomes using microfluidic hydrodynamic focusing facilitates self-assembly of liposomes when mixing lipids dissolved in an organic solvent with an aqueous buffer by lamellar mixing [6,7]. One hypothesis is that the liposomes are formed initially from organic solvent-stabilized lipid discs, which bend and close to globules upon the subsequent depletion of the organic solvent [8]. Thus, this method is a bottom-up method, as the liposomes are assembled from monomeric lipids and the final liposomes are the largest structure in the process. Some physicochemical characteristics, particularly the particle size, of liposomes prepared by microfluidics can be influenced by the variables; total flow rate (TFR), flow rate ratio (FRR) between the two phases, choice of aqueous buffer and organic solvent, and lipid concentration in the organic solvent [7,9]. The microfluidics method is suitable to prepare liposomes complexing nucleic acids, either formulated from preformed liposomes mixed with, e.g., RNA or DNA [10,11] or by directly complexing the nucleic acids during liposome formation [12]. In the latter situation, the result might be formation of lipid nanoparticles rather than liposomes, due to the interior complexation of nucleic acids with the cationic lipids [12].

An important difference between the two manufacturing methods is the homogeneity of the conditions each lipid molecule experiences during the formulation process. When liposomes are prepared by microfluidics, the continuous unidirectional movement of the formulation components within the narrow channel facilitates reproducible conditions across the stream independent of lapsed time [4], and consequently each lipid molecule will experience very similar conditions during the self-assembly into liposomes. In contrast, during the rehydration of a dry lipid film by high shear mixing, the lipid molecules may experience very different conditions depending on their location in the sample, e.g., the distance from the shearing tool or position within the dry lipid bilayer in relation to the water interphase. This reduces control of process, possibly resulting in larger and more heterogeneous particle suspensions.

The microfluidic method has some manufactural advantages as compared to the lipid film rehydration methods used for liposomes. Importantly, the microfluidics method is, in contrast to the lipid film rehydration methods, easily scalable and can be set up for continuous production [13,14]. Furthermore, the microfluidics method is often reported to result in highly controlled and reproducible particle sizes and with low polydispersity indexes, a feature that can be very important in manufacturing as it can have great impact on both stability and functionality of the formulation [15,16,17]. Depending on the adjustment of the different process parameters, the microfluidics method can often be optimized to produce liposomes with particle sizes less than 100 nm [9,12,18,19]. This is an attractive feature for CAF09b, which when produced by high shear mixing, has an average particle size of 200–250 nm [1,3].

In the present study, we evaluate the effect of varying microfluidic process parameters on resulting physicochemical characteristics of CAF09b. The process parameters evaluated are the EtOH concentration (FRR), lipid concentration, and dimethyl sulfoxide (DMSO) concentration, which were incorporated into a design of experiments (DoE) study to investigate how variation of the parameters interact. The aim was to establish which combinations of the variable parameters that result in colloidally stable particles with acceptable particles sizes, i.e., ideally less than 100 nm. The ability to induce CTL responses by CAF09b prepared by the microfluidics method and HSM was assessed to find out whether the novel manufacturing method altered the CAF09b adjuvanticity.

When employing a novel manufacturing method, it is necessary to not only evaluate the physicochemical parameters but also to consider if the induced immune responses are impacted upon. For CAF09b, the most relevant correlate of immunity is the antigen-specific CD8^+^ T-cell response after i.p. immunization [1]. Therefore, this administration route was used to compare the ability to induce CD8^+^ T-cell responses by CAF09b prepared by the microfluidics and HSM methods.

## 2. Materials and Methods

### 2.1. Materials

Dimethyldioctadecylammonium bromide and monomycoloyl glycerol analogue 1 were obtained from NCK A/S (Farum, Denmark) and poly(I:C) was purchased from Sigma-Aldrich (St. Louis, MO, USA). All other chemicals were used at analytical grade and purchased from commercial suppliers.

### 2.2. Preparation of Liposomes Using Microfluidics

Weighed amounts of DDA and MMG were dissolved in EtOH 99%, while polyI:C and, if required, DMSO were diluted in Tris-buffer (10 mM, pH 7.4). The liposomes were prepared by using a NanoAssemblr^TM^ Benchtop with NanoAssemblr software v. 1.0.8 (Precision NanoSystems, 2018, Vancouver, Canada) at a TFR of 12 mL/min. The FRR, [Lipid] in the organic phase and [DMSO] were varied according to the design of experiments protocol (Table 1). The formulations were directly diluted by dispensing them from the chip into a vial pre-filled with Tris-buffer, and the final formulations contained 2.5/0.5/0.125 mg/mL DDA/MMG/poly(I:C). Samples in the DoE study were split in two and stored at either room temperature (RT) or at 4 °C.

### 2.3. Preparation of Liposomes with High Shear Mixing

For comparison in the in vivo vaccination studies, CAF09b was prepared with the HSM method, essentially as described elsewhere [3]. Briefly, weighed amounts of DDA and MMG were dissolved in EtOH and dried overnight in a vial under a gentle N_2_ stream. The lipid film was rehydrated in Tris-buffer at 60 °C for 15 min using a Heidolph Silent Crusher equipped with a 6F shearing tool (Heidolph Instruments GmbH, Schwabach, Germany) at 26,000 rpm. Subsequently, the poly(I:C) was slowly added over 30 min by using a pump, while maintaining the mixing conditions. The final formulations contained 2.5/0.5/0.125 mg/mL DDA/MMG/poly(I:C).

### 2.4. Physicochemical Characterization of Liposomes

The average hydrodynamic diameters (*z*-average), polydispersity indexes (PDI) and zeta potentials of the liposomes were determined on a Zetasizer Nano ZS (Malvern Instruments Ltd, Worcestershire, UK) equipped with a 633 nm laser and 173° detection optics. The samples were diluted 10 times in milliQ water prior to measurement, and the complex buffer function in the instrument software was used to calculate the viscosity and refractive indexes based on the residual [EtOH]. Zetasizer Software v7.12 (Malvern Instruments Ltd) was used for acquisition and analysis of the software.

### 2.5. Quantification of Poly(I:C)

The recovery of poly(I:C) from CAF09b formulations was evaluated using the Quant-iT^TM^ Ribogreen^TM^ assay (ThermoFisher Scientific Inc., Waltham, MA, USA) essentially according to the manufacturer’s manual. The CAF09b samples were assayed either untreated or after treatment with 1% *w/v* sodium dodecyl sulfate (SDS, Sigma-Aldrich). The samples were diluted 100× in RNAse free TE-buffer and a 100 µL of sample or poly(I:C) standard were mixed with 100 µL Ribogreen reagent in a black 96-well plate. The samples were analyzed using a Polarstar Omega (BMG Labtech, Ortenberg, Germany) at excitation/emission wavelengths 485/520 nm.

The release of poly(I:C) from CAF09b by treatment with varying concentrations of SDS was evaluated using agarose gel and detection with ethidium bromide. The agarose (Sigma-Aldrich) was dissolved in TAE-buffer (Tris-acetate 40 mM, 1 mM EDTA, pH 8.0) and ethidium bromide was added at a concentration of 0.2 µg/mL gel. The CAF09b and poly(I:C) standard was diluted to an equivalent of 1 µg poly(I:C) in Tris-buffer with 17% *v/v* glycerol and a final concentration of SDS ranging from 0–3% *w/v*. The samples were run in the gel for 20 min at 100 V, and the gel was visualized using a UV table VWR Genosmart (VWR, Radnor, PA, USA).

### 2.6. Immunogenicity Studies

The adjuvanticity of CAF09b prepared by the microfluidics or HSM method were compared. The in vivo studies were conducted at Statens Serum Institut in accordance with regulations imposed by the national animal protection committee and European Community Directive 86/609. The study design was approved by the governmental Animal Experiments Inspectorate under license 2017-15-0201-01363. Groups of six female, 7–9-week-old C57BL/6 mice (Envigo, Horst, the Netherlands) were immunized intraperitoneally with 10 µg/dose of a 15-mer peptide containing the CD8 epitope RAHYNIVTF from HPV-16 E7 (JPT, Berlin, Germany), without adjuvant as a negative control or adjuvanted with CAF09b prepared by microfluidics (CAF09b MF) or high shear mixing (CAF09b HSM). The adjuvant doses were 250/50/12.5 µg/dose DDA/MMG/poly(I:C) in a total volume of 200 µL. The vaccine was prepared by admixing the HPV-16 E7 peptide antigen with CAF09b over 30 min with intermittent vortexing to allow electrostatic adsorption of the peptide onto CAF09b. The vaccines were administered within 2 h of admixing. The mice were immunized twice with a two-week interval and the immune responses were assessed one week after the final immunization.

The blood, spleens and draining mediastinal and tracheobronchial lymph nodes (LNs) were removed and processed to single cell suspensions, the blood by treatment with Lympholyte (Cedarlane, Burlington, CA, USA) and the organs by passing through a nylon mesh cell strainer (Corning Inc. Corning, NY, USA). The cells were subsequently washed with PBS and RPMI 1640 (Invivogen, San Diego, CA, USA). 

The frequencies of antigen-specific CD8^+^ T cells in blood, spleens and LNs were analyzed by using multimer flow cytometry. The cells (10^6^ cells/well) were stained with H2-D^b^-RAHYNIVTF:PE (ProImmune, Oxford, UK), anti-mouse CD19:PerCP-Cy5.5 antibody (Ab, 1D3) and CD4:eFluor780 Ab (RM4-5) from eBiosciences (San Diego, CA, USA), and CD8:BV421 Ab (53-6.7), CD44:APC Ab (IM-7), and CD62L:FITC Ab (MEL-14), all from BD Biosciences (San Jose, CA, USA). Data were acquired by using a BD Fortessa flow cytometer (BD Biosciences) and analyzed by using the FlowJo vX software (Tree Star, Ashland, OR, USA) identifying immune cell subsets.

Splenocytes (2 × 10^5^ cells/well) were stimulated with 2 µg/mL RAHYNIVTF peptide (JPT, Berlin, Germany) in RPMI 1640 supplemented with 10% (*v/v*) heat-inactivated fetal calf serum, 5 × 10^−6^ M β-mercaptoethanol, 1% (*v/v*) penicillin-streptomycin, 1% (*v/v*) sodium pyruvate, 1 mM L-glutamine, and 10 mM HEPES (cRPMI), as described elsewhere [2]. Cells incubated with cRPMI medium or 3 µg/mL Concanavalin A (Sigma Aldrich) were used as negative and positive controls, respectively. The cells were incubated at 37 °C and 5% CO_2_ for three days, then the supernatants were harvested and analyzed for IFN-γ levels using ELISA. Briefly, Maxisorp plates (Nunc, Hillerød, Denmark) were coated with capture IFN-γ antibody (BD Biosciences) overnight before being blocked with 2% skim milk in PBS. Diluted supernatants in PBS with 2% bovine serum albumin (BSA) were added to the wells along with IFN-γ standards and incubated at room temperature (rt) for 2 h. The samples were incubated with biotin-conjugated anti-mouse IFN-γ detection Ab (BD Biosciences) for 1 h, followed by streptavidin-conjugated horseradish peroxidase incubation for 30 min. Detection was performed with 3,3′,5,5′-tetramethylbenzidine (Kem-En-Tec, Taastrup, Denmark), and the reaction was stopped with 0.2 M H_2_SO_4_. The optical density was read at 450 nm with 570 nm correction.

### 2.7. Statistical Analysis

The DoE investigating the interaction of selected process variables for the microfluidics method (Table 1) were evaluated with the particle size on day of manufacture (day 0) and on day 1, 7, 14 and 21 with the corresponding particle size ratios calculated in relation to the particle sizes at day 0, zeta potential on day 0, and poly(I:C) quantification on day 7. The results of the DoE were analyzed using face-centered response surface methodology (RSM) with backward elimination using Minitab 18.1 (Minitab, Inc, State College, PA, USA). The quality of the models was assessed based on the adjusted R^2^ and lack-of-fit, and if these were unacceptable, the model was rejected. The particle size ratios were additionally modeled for [DMSO] and [Lipid] for each of the three [EtOH] levels using 2-parameter response surface modeling. For all models in the manuscript, the statistical significance of each parameter is stated in Table S1.

## 3. Results

### 3.1. The Concentration of EtOH Significantly Affected the Particle Sizes, Polydispersity and Zeta Potentials of Freshly Prepared Formulations

Manufacture of CAF09b by the high shear mixing method is a several step process performed in batches involving the formation of a dry lipid film, a rehydration step to prepare the liposomes and a subsequent step for addition of poly(I:C) [1]. Furthermore, the lipid film rehydration and addition of poly(I:C) has to be done at elevated temperatures due to the high phase transition temperature of DDA (approximately 45 °C) [20]. A GMP production would benefit from the ability to easily scale manufacture in a batch-independent manner and to be able to keep the entire process at ambient temperature. Therefore, microfluidics pose a promising manufacturing method for establishing a one-step process for the preparation of CAF09b.

The liposomal adjuvant CAF09b could be formulated with the microfluidics method in a one-step process, and the concentration of EtOH, the lipid concentration and the DMSO concentration were shown to influence the resulting particle sizes and colloidal stability, measured as change in particle size, in relation to storage conditions. The TFR, on the other hand, was found not to affect the physicochemical characteristics of the liposomes. This is in accordance with a study applying microfluidics to formulate a variety of liposomes composed of different lipids, which showed that the FRR was the main factor affecting the particle size, whereas TFR mainly had an impact on the speed of manufacture [21].

Therefore, a design of experiments was set up to investigate how these variables co-affect the colloidal stability, zeta potential, and association of poly(I:C) of the resulting particles (Table 2). The parameters were varied as described in Table 1, and all possible combinations with six repeats at the center point were prepared, resulting in 32 different samples. The samples were physicochemically characterized measuring the average particle sizes (*z*-average), polydispersity indexes, and zeta potentials on the day of manufacture. A face-centered RSM was chosen for DoE analysis because it covered the whole design space and incorporate curvature in the responses, which could not be further expanded as i) the minimum level of [DMSO] was 0% and ii) the high level of [Lipid] could not be further increased, as pilot studies had indicated that this would cause aggregation and clogging of the microfluidic chip. For each modeled response, graduated plots were established indicating intervals of changes across the levels of the applied parameters with significant effect (Table of significance of parameters in the RSM models; Table S1). The quality of the model was expressed as lack-of-fit, which should have *p* > 0.05 and adjusted *r*^2^, which should approach 100%. The plots may be used to identify patterns in the responses as a function of the parameter levels.

**Figure 1:**
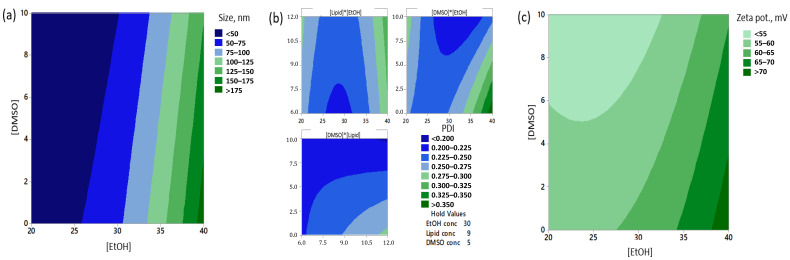
Modeling of physicochemical data for formulations at the day of manufacture (day 0) from design of experiments were analyzed using face-centered response surface methodology to evaluate the effects of the parameters [EtOH], [Lipid], and [DMSO]. Contour plots for the read outs for (**a**) particle size, lack-of-fit *p* = 0.177, adj. *r*^2^ = 97.62%, (**b**) polydispersity indexes (PDI), lack-of-fit *p* = 0.044, adj. *r*^2^ = 66.84%, and (**c**) zeta potential, lack-of-fit *p* = 0.962, adj. *r*^2^ = 46.35%.

It was possible to use RSM to model the effect of the variables on the particle sizes of the freshly prepared formulations (Figure 1a). Thus, the concentration of EtOH was found to be highly significant for the particle sizes, and displayed curvature ([EtOH]*[EtOH], *p* ≤ 0.0001). The [DMSO] was also significant and interacted with the [EtOH], with high [EtOH] and [DMSO] resulting in the smallest particle sizes. The RSM model for the corresponding PDI values showed significant lack-of-fit, indicating the data do not fit the suggested model fully (Figure 1b), however, the PDI model has been included to complement the particle size model. It does appear that in addition to the [EtOH] and [DMSO], the [Lipid] may affect the resulting PDI values.

Though all formulations were highly cationic, variations in the zeta potentials were observed, which were directed by the [EtOH] and [DMSO] (Figure 1c). Thus, it appears that the concentration of EtOH (equivalent to the FRR) and the concentration of DMSO are the main factors impacting the evaluated physicochemical characteristics of CAF09b when manufactured via microfluidics.

### 3.2. Addition of DMSO Stabilized CAF09b Formulations Stored at 4 °C

**Figure 2:**
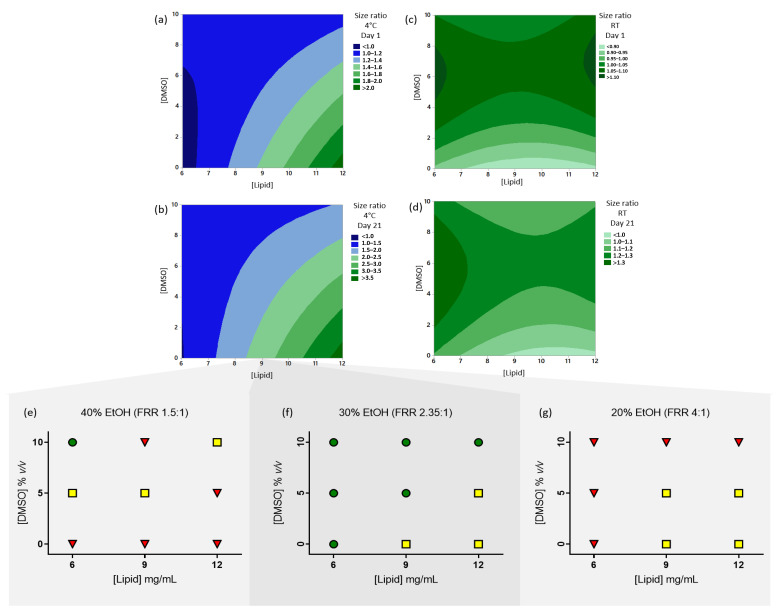
The data from design of experiments were analyzed using face-centered response surface methodology to evaluate the effects of the parameters [Lipid] and [DMSO] at a fixed [EtOH] of 30% *v/v*. Contour plots for the size ratios at (**a**) day 1, storage at 4 °C, lack-of-fit *p* = 0.320, adj. *r*^2^ = 63.68%, (**b**) day 21, storage at 4 °C, lack-of-fit *p* = 0.145, adj. *r*^2^ = 72.73%, (**c**) day 1, storage at room temperature (RT), lack-of-fit *p* = 0.179, adj *r*^2^ = 20.88% and (**d**) day 21, storage at room temperature (RT), lack-of-fit *p* = 0.081, adj. *r*^2^ = 18.93%. Particle stability of samples stored at 4 °C for 21 days was evaluated qualitatively. Samples prepared at (**e**) 20% EtOH (FRR 4:1), (**f**) 30% EtOH (FRR 2.35:1) and (**g**) 40% EtOH (FRR 1.5:1) after 21 days stored at 4 °C. Green circles: particle sizes ≤100 nm, yellow squares: 100–250 nm, red triangles: >250 nm.

When CAF09b was prepared by microfluidics, the storage conditions were shown to affect the colloidal stability of the liposomes. Thus, the particle sizes of pilot study samples analyzed one day after preparation did not change for formulations stored at RT, whereas the particle sizes would increase when they were stored at 4 °C. However, it was shown that addition of DMSO to the Tris-buffer diminished the changes in particle sizes upon storage at 4 °C. The colloidal stability of the formulations in the present study were assessed as the ratio of the particle sizes measured upon storage to the particle sizes of the freshly prepared formulations. It was not possible to do RSM modeling of the particle size ratios for all parameters at once, but when the [EtOH] was kept constant at 30%, it was possible to model the [Lipid] and the [DMSO] (Figure 2). Both parameters significantly affected the changes in particle sizes for formulations stored at 4 °C, with high concentrations of lipid and low [DMSO] conferring low colloidal stability at both day 1 and day 21 after manufacture (Figure 2a,b). In the RSM model, the pattern of changes in particle sizes were similar at day 1 and day 21, indicating there were no changes in the development of particle sizes after the initial increase. Therefore, DMSO had a stabilizing effect on the liposomes at 4 °C storage. The RSM models of the particle size ratio of formulations stored at RT has low predictable value (Figure 2c,d), but there was not the same stabilizing effect of DMSO as was observed for formulations stored at 4 °C. However, the pattern of changes in the particle size ratio was similar at day 1 and day 21 for samples stored at RT, indicating that the mechanisms of particle size changes were similar with time. Interestingly, it appeared that the mechanisms of particle size changes were very different depending on the storage temperature, which may be due to temperature induced differences in how the lipids and poly(I:C) within the particles interact.

### 3.3. Formulations Prepared with 30% v/v EtOH Have Most Samples at Less Than 100 nm after Storage at 4 °C for 21 Days

One aim of the study was to establish the process parameters that resulted in particle sizes at less than 100 nm, which are optimal for delivery of vaccine to draining lymph nodes and the spleen. The particle sizes for formulations stored at 4 °C for 21 days were plotted in a heat-map like style to give an overview of the process parameters that resulted in particle sizes of less than 100 nm (green circles), 100–250 nm (yellow squares) and more than 250 nm (red triangles) (Figure 2e–g). Formulations prepared with 30% *v/v* EtOH (FRR 2.35:1) resulted in most samples with particle sizes less than 100 nm as compared to using 20% *v/v* or 40% *v/v* EtOH, with lower [Lipid] and higher [DMSO] promoting this (Figure 2e–g). In contrast, formulations prepared with 20% *v/v* EtOH showed formulations at 100–250 nm when formulated with high [Lipid] and lower [DMSO], while other samples were larger than 250 nm (Figure 2e–g). 

### 3.4. Poly(I:C) Is Completely Encapsulated Inside the Liposomes after Manufacture by Microfluidics

**Figure 3:**
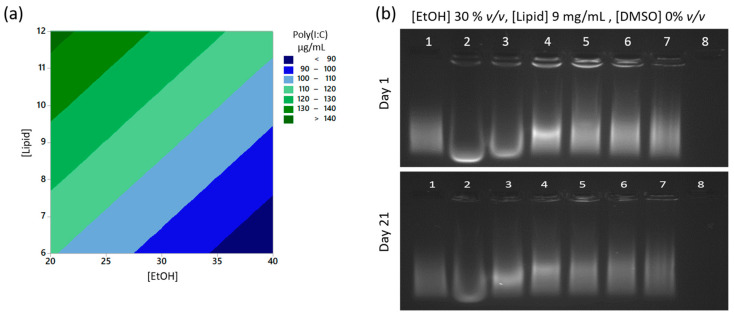
Poly(I:C) (125 µg/mL) in samples treated with 1% *w/v* SDS or untreated was quantified with the Ribogreen assay. Poly(I:C) could not be detected in untreated samples. (**a**) RSM model of the amount of poly(I:C) in samples stored at 4 °C for 7 days, lack-of-fit *p* = 0.082, adj. *r*^2^ = 44.03%. (**b**) A representative sample (sample 13) was treated with 0, 0.1, 0.25, 0.5, 1, 2, and 3% SDS, and analyzed with agarose (1%) gel electrophoresis the day after preparation and 21 days after preparation, storage at 4 °C. Lane 1: Poly(I:C) control, lane 2–8: CAF09b treated with 3, 2, 1, 0.5, 0.25, 0.1 and 0% SDS, respectively. Three other representative samples are depicted in Figure S2. The particle sizes, PDIs, and size ratio of samples at day 7 stored at 4 °C and the RSM model of the size ratio of the samples prepared at 30% EtOH is shown in Figure S1.

We wished to establish whether the poly(I:C) in the formulations could be recovered after the microfluidics manufacturing procedure. The concentration of poly(I:C) in the samples was determined using the Ribogreen assay, evaluating both untreated samples and samples treated with SDS, 1% (*w/v*) after storage at 4 °C or RT for 7 days. In the Ribogreen assay, the reagent binds within the dsRNA resulting in a fluorescent signal. The levels of poly(I:C) were below the detection limit for all untreated samples, indicating the fluorescent dye was not able to interact with the poly(I:C) in the CAF09b formulations. The samples were subsequently treated with the negatively charged SDS, which disrupts the liposomes and can displace nucleic acids by binding to the positively charged lipid headgroups [22]. Different levels of poly(I:C) were detectable in the samples treated with SDS (averaging 116 ± 22 µg/mL for samples stored at 4 °C, and 103 ± 36 µg/mL for samples stored at RT, corresponding to 93 ± 18% and 83 ± 29% of the theoretical concentration, respectively). It was possible to use face-centered RSM to model the poly(I:C) content in samples stored at 4 °C for all variables. The [EtOH] and [Lipid] were found to significantly affect the levels of detected poly(I:C) in SDS treated samples stored at 4 °C (Figure 3a), with higher levels of poly(I:C) detected at high [Lipid] and low [EtOH]. Thus, it is the samples with the lowest residual EtOH content that display the highest levels of recoverable poly(I:C), which may imply that the EtOH denatures or in other ways affects the poly(I:C). The RSM modeling of the size ratio of the samples at day 7 stored at 4 °C and prepared at [EtOH] 30% *v/v* showed a similar pattern as seen for the size ratio at day 1 and day 21 (Supplementary Figure S1). Thus, any possible effects of the particle sizes on poly(I:C) loading are likely to be constant with time. The levels of poly(I:C) detected in SDS treated samples stored at RT could not be modeled.

From the Ribogreen assay it was not possible to tell whether poly(I:C) was encapsulated inside the liposomes or if some of it was adsorbed to the liposomal surface. Therefore, representative samples were qualitatively evaluated by agarose gel electrophoresis using ethidium bromide to visualize the poly(I:C). Sample 13 ([EtOH] 30% *v/v*, [Lipid] 9 mg/mL, [DMSO] 0%, *v/v*) was chosen as a representative sample, and release of poly(I:C) was evaluated at day 1 and 21 after manufacture (Figure 3b). Other samples were chosen to evaluate the effect of adding DMSO (samples 13 and 20), furthermore, sample 20 was colloidally stable at both 4 °C and RT for 21 days. Sample 1 was colloidally unstable at both 4 °C and RT, whereas sample 24 was colloidally stable at RT but unstable at 4 °C (Figure S2).

All samples had similar patterns of poly(I:C) release after SDS treatment. No poly(I:C) could be detected in the untreated samples the day after preparation stored at 4 °C and RT, while progressively more poly(I:C) was detectable at treatment with increasing concentrations of SDS (0.1–3%) (Figure 3b, Figure S1). Treatment with just 0.1% SDS induced detection of poly(I:C) inside the liposomes in addition to detection of released poly(I:C) in the gel. At concentrations of 2 and 3% SDS, almost all poly(I:C) appeared to have been displaced from the liposomes. This was determined based on the poly(I:C) signal detected in the wells, which was very low for the samples treated with 2 or 3% SDS, respectively. For the samples treated with 0.1–1% SDS a stronger signal was present in the well. Hence, poly(I:C) could be detected by ethidium bromide while still associated to CAF09b, evident from the signals observed in the wells of SDS treated samples. This indicates no poly(I:C) was adsorbed to the surface of the liposomes as there was no detectable signal in the untreated samples. Thus, the gel electrophoresis results confirm that poly(I:C) is completely complexed inside the liposomes and therefore inaccessible for the detecting agent ethidium bromide.

The pattern of poly(I:C) detection and displacement did not change upon storage for 21 days, except poly(I:C) was detectable in the SDS untreated sample 1, which was highly aggregated (approx. 4000 nm) at that point. Thus, poly(I:C) does not appear to change the pattern of association with DDA upon storage.

### 3.5. Antigen-Specific CD8^+^ T-Cell Immune Responses Induced by CAF09b Prepared by the Microfluidics or High Shear Mixing Methods Are Comparable

Lipid rehydration by high shear mixing has until now been the manufacturing method of choice to prepare CAF09b, whereas we have shown in the present paper that preparation by microfluidics resulted in acceptable formulations. However, comparing the immune responses induced by CAF09b prepared by the two manufacturing methods is necessary to evaluate whether they are interchangeable for manufacture of vaccine adjuvants.

**Figure 4:**
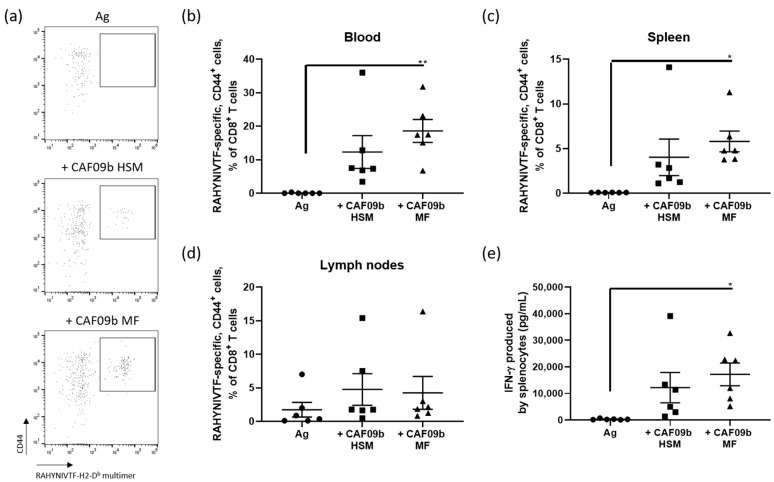
Evaluation of the CD8+ T-cell responses induced by CAF09b prepared by either the lipid film rehydration or microfluidics methods. Groups of six female C57BL/6-mice were immunized intraperitoneally (i.p.) twice with two-week-intervals with 10 µg/dose 15-mer peptide containing the HPV-16 E7 minimal epitope RAHYNIVTF either unadjuvanted or adjuvanted with CAF09b prepared by the high shear mixing (CAF09b HSM) or the microfluidics (CAF09b MF) methods. (**a**) The antigen-specific CD8^+^ T-cell responses (defined as CD19^−^, CD4^−^, CD8^+^) were analyzed as CD44^+^, RAHYNIVTF-H2-D^b^-PE multimer^+^ by flow cytometry, representative samples of the responses in each group in the blood. The analyses were performed in (**b**) the blood, (**c**) spleen, and (**d**) draining mediastinal and tracheobronchial lymph nodes. (**e**) Production of IFN-γ by splenocytes stimulated with RAHYNIVTF analyzed by ELISA. *n* = 6, bars represent mean ± SEM. * *p*≤ 0.05, ** *p* ≤ 0.01.

A 15-mer peptide of the cervical cancer protein, HPV-16 E7, which contains the CD8 epitope RAHYNIVTF, was used as a model antigen. The antigen was administered twice with two-week-intervals intraperitoneally (i.p.) to female C57BL/6 mice either unadjuvanted as negative control or adjuvanted with CAF09b prepared by either high shear mixing (CAF09b HSM) or microfluidics (CAF09b MF). The CAF09b MF, sample 32 (Table 2) with 12 mg/mL lipids, 20% *v/v* EtOH and 10% *v/v* DMSO was used to reduce the residual EtOH content as much as possible (2.5% *v/v* in the final vaccine), while DMSO stabilized the formulations. New CAF09b formulations were prepared a couple of days prior to each immunization and stored at 4 °C. The CAF09b formulations were used within seven days, where complete poly(I:C) loading was shown, while admixing of the adjuvant and antigen was performed within two hours of administration. The CAF09b formulations were therefore assumed to retain full adjuvant potential. One week after the final immunization the mice were euthanized and the spleen, draining lymph nodes (the tracheobronchial and mediastinal lymph nodes), and blood were harvested, and the levels of antigen-specific CD8^+^ T cells were analyzed by RAHYNIVTF-H2-D^b^ multimer flow cytometry (Figure 4a). 

There were no significant differences between the immune responses induced by CAF09b prepared by the two different manufacturing methods (Figure 4). Only CAF09b MF was significantly different from the unadjuvanted HPV-16 E7 peptide control, whereas CAF09b HSM did not induce a significant immune response in the blood and spleen (Figure 4b,c). Adjuvanting the antigen with CAF09b MF or CAF09b HSM did not induce significantly more antigen-specific CD8^+^ T cells in the draining LNs as compared to unadjuvanted antigen (Figure 4d). There was no significant difference in the IFN-γ levels between the CAF09b HSM and CAF09b MF, but CAF09b MF was significantly higher than the unadjuvanted antigen group (Figure 4e).

## 4. Discussion

Manufacture of liposomal formulation by using microfluidics has potential benefits for large-scale production as compared to the standard lipid rehydration methods, as it is readily scalable and reduces loss of material [23]. In the present study, we have shown that it is possible to manufacture CAF09b by using hydrodynamic focusing microfluidics, with the resulting particles in the nanometer range that were colloidally stable depending on the process parameters. Acceptable physicochemical characteristics were achieved within a range of process parameters, thus CAF09b formulated with 30% EtOH at lipid concentrations of 6–9 mg/mL and 5–10% DMSO resulted in liposomes remaining at <100 nm at 21 days after manufacture. Thus, we have identified a design space within which it is possible to manufacture formulations with acceptable physicochemical properties. This allows for further optimization of the CAF09b manufacture protocol based on other parameters and may be used as a guideline for manufacture optimization of similar formulations.

It was not possible to use RSM to model the changes in particle sizes at storage, which indicates the aggregation pattern does not follow a set model. Furthermore, the measurement of large and polydispersed particle suspensions becomes imprecise when using DLS. Therefore, the deviation in the measurements from the actual particle sizes might interfere with the modeling. However, it was possible to model the changes in particle size relative to the initial sample sizes when keeping [EtOH] constant at 30% *v/v*. This emphasizes the substantial impact the [EtOH] has on the formation and stability of the liposomal particles.

The concentration of EtOH is the process parameter, which had the greatest influence on the physicochemical formulation outcomes, such as particle size, zeta potential, and colloidal particle stability. This is in accordance with several publications showing that altering FRR enables precise tuning of the resulting particle sizes. Generally, increased concentrations of EtOH results in an increase in the particle sizes, though the exact particle sizes achieved also depend on the microfluidic system, and the type and concentration of lipids and other components [6,21,24,25]. The RSM modeling showed a positive correlation between the [EtOH] and initial particle sizes (Figure 1a). This might be due to the mechanics of liposome formation from lipid discs, as the critical precipitation concentration of the lipids will be reached quicker at low [EtOH] [7]. Thus, there will be a limited time for disc formation before they close up to form liposomes, which will then be of limited size.

In the formulations prepared in the present paper, the EtOH was not removed after liposome formation. Rather, the formulations were prepared at a sufficiently high lipid concentration that enabled dilution of the liposomes to a final concentration of the adjuvant of 2.5/0.5/0.125 mg/mL DDA/MMG/poly(I:C), which is the concentration usually used for CAF09b analogues [3]. The resulting EtOH concentrations in the formulations were 5–20% depending on the initial [Lipid] and FRR. The presence of EtOH in the final formulations might have had an effect on the colloidal stability of the liposomes, as it would be expected to interact with the lipid bilayers and possibly destabilize them. Phospholipid bilayers were destabilized by increasing concentrations of EtOH and the bilayer thickness was reduced [26]. Furthermore, ethanol may interact with the lipid bilayers resulting in interdigitation induced by EtOH insertion in the bilayer close to the head-groups, which has been shown for a range of phospholipids [27,28,29]. This in turn affects the surface area of each lipid within the bilayer, which increases at addition of EtOH accompanied by a decrease in bilayer thickness [29]. These effects may also be present in CAF09b, which might contribute to the reduced colloidal stability observed at high [EtOH].

The choice to add DMSO to the aqueous phase of CAF09b was based on studies with phospholipid-based liposomes, which showed that DMSO has a stabilizing effect on the lipid membrane [30,31]. This includes direct interaction with the lipid headgroups and carbon chains, which promotes stabilization in the gel phase and possibly formation of bilayers rather than interdigitated membranes [31,32]. We hypothesized that the stabilizing effect of the lipid membrane would translate to a stabilizing effect on the particle sizes at increased DMSO content, as the probability of membrane fusion and aggregation might be lowered. Indeed, addition of DMSO in the aqueous phase had a significant correlation with the colloidal stability of the particles.

The particle sizes were the physicochemical characteristic that was most varied by using different microfluidic parameters. Particle sizes have been reported to have substantial impact on the induced immune responses [33,34,35,36], which can be attributed to the effect on the biodistribution patterns and cellular uptake mechanisms [37,38]. Particles at <200 nm may be able to drain away from the injection site [39], and larger particles are more susceptible for uptake by phagocytic cell populations such as macrophages [37]. However, in the particular case of i.p. vaccination with cationic particles, the initial particle sizes are not expected to have a significant impact on the induced immune responses as they will likely aggregate immediately upon contact with interstitial proteins [40]. Thus, the particles encountered by the immune cells in the peritoneal cavity will likely be vastly different from the particles in the formulation.

While the particle sizes might not have an impact on the adjuvanticity of CAF09b, it is useful as a parameter for manufacturing control. An often-evaluated quality of hydrodynamic focusing microfluidics is the ability to precisely and reproducibly engineer particles in the nanometer range [6,12,13,41]. This may be used as a control parameter for manufacturing and scale-up studies, as any great variation from the expected particle sizes might indicate a faulty formulation. Reducing the particle sizes might also have a positive effect on the colloidal stability and might thus prolong shelf life; smaller particles might not sediment as quickly as larger particles and aggregates.

The adjuvanticity of CAF09b prepared by microfluidics was compared to preparation by the previously used lipid film rehydration by high shear mixing method. CAF09b prepared by the two methods appeared to induce similar CD8^+^ T-cell responses against the model antigen following i.p. administration (Figure 4). The immunogenicity of the HPV-16 E7 peptide antigen was not altered by the formulation method of CAF09b. Thus, the level of immune responses observed in the present study was comparable to previous studies with HPV-16 E7 adjuvanted with CAF09 [1]. It was expected that the adjuvanticity of the two CAF09b formulations were similar, as CAF09b MF maintained the high cationic surface charge, which has been identified as a very important physicochemical characteristic for adjuvanticity [42], as the cationic charge enables high loading efficiencies of the antigen and poly(I:C) by electrostatic interactions. Most protein and peptide antigen have isoelectric points at approximately 4–6, which have been shown to readily adsorb to the surface of different CAF formulations upon admixing [42,43,44], due to the opposite charges of the cationic DDA and anionic antigen at neutral pH. The theoretical pI of the HPV-16 E7 peptide antigen used in this study is 5.38, and it will therefore adsorb to a similar high level to CAF09b prepared by either HSM or MF. While the [DMSO] and [EtOH] had an effect on the measured zeta potentials, they remained highly cationic across all prepared formulations (Figure 1c). The observed differences in the measured zeta potentials depending on the [DMSO] and [EtOH] might be explained by considering how the zeta potential is defined; as the thickness of the layer of structured water molecules surrounding a charged particle. Possibly, the interaction of DMSO and EtOH with the surface of the lipid membrane, but also the mode of incorporation of dissolved molecules within the water lattice, affects the thickness and stability of the structured water layer. The samples were diluted in mQ-water prior to zeta potential measurement, which presumably will reduce the contribution from dissolved organic solvents.

The cationic liposomal adjuvant, CAF01, containing DDA and trehalose-6,6-dibehenate was prepared by both the microfluidics and lipid film rehydration methods [45]. The immune responses to the recombinant *M. Tuberculosis* antigen H56 adjuvanted with the two CAF01 formulations were compared in C57BL/6 mice. Antigen-specific IgG1 and IgG2c responses were similar for mice vaccinated with the two CAF01 formulations. In the spleens, antigen-specific stimulation of IL-17 and IFN-γ production were also similar between the CAF01 formulations, whereas a significantly higher IL-17 and IFN-γ production was stimulated in the draining lymph nodes after immunization with H56 adjuvanted with CAF01 manufactured by the microfluidics method. Thus, the adjuvanticity of CAF01 appears to be retained or even slightly improved upon manufacture by the microfluidics method. This indicates that altering the manufacturing method does not compromise the adjuvanticity of CAF adjuvants.

In this paper, we have shown that it is possible to prepare the complex formulation CAF09b in a single-step process by using hydrodynamic focusing microfluidics. In the protocol, the concentration of EtOH was reduced by diluting the samples with Tris-buffer after formulation. EtOH is a class 3 solvent according to the ICH Q3C-R6 guideline, and may be administered at a dose of 50 mg/day without further justification [46]. The CAF09b dose in clinical trials is up to 1000 µL. Thus, only some samples prepared in this study complied with the ICH guidelines, as the EtOH content after dilution was 5–20% *v/v*. However, it would still be beneficial to remove residual EtOH during GMP manufacture. This may be achieved by dialysis, which has the disadvantages of being time-consuming and is often not applicable for continuous production. An alternative method for solvent removal and exchange is tangential flow filtration, which can be incorporated into a continuous manufacturing protocol [19,47]. However, application of tangential flow filtration requires evaluation of the filters used, as they have to be compatible with all components in the formulation in question to avoid, e.g., clogging. Another hindrance for transferring CAF09b manufacture to GMP production is the requirement of DMSO in the aqueous buffer to stabilize the liposomes. The effects of DMSO on lipid membranes, which stabilize the liposomes in formulation, may cause adverse effects on the cells at the injection site upon administration. Therefore, it is necessary to identify an alternative to DMSO for liposome stabilization in a GMP-approved formulation. Finally, the sterility of the product must be considered. Terminal sterilization of a formulation is preferred. However, even though several of the CAF09b samples prepared by microfluidics had particles sizes less than 200 nm, sterile filtration is not an obvious option due to the cationic nature of CAF09b. An alternative is γ-irradiation, which has been applied to CAF01, however the adjuvant was freeze-dried prior to sterilization [48]. Therefore, another strategy may be aseptic production, which requires that the components are sterile prior to commencement of manufacture and the manufacturing equipment is sterilized. A possibility for microfluidic GMP production is to utilize pre-sterilized single-use parts, which are in direct contact with the formulation, e.g., the microfluidic chip, tubing and containers.

## 5. Conclusions

The manufacturing methods of liposomes have traditionally relied on different lipid rehydration methods, which are difficult to scale up for commercial production. This complicates the transfer of promising liposome-based adjuvants to the clinic, and therefore scalable manufacturing methods may ease the process of preparing novel formulations for large-scale manufacture.

We successfully transferred the manufacture of CAF09b from a complex and time-consuming multi-stage protocol to a microfluidic-based single-step liposome synthesis process. Satisfactory physicochemical characteristics could be achieved within a window of parameter levels, namely particle size and colloidal stability. Thus, we found that especially the FRR was an important determinant of the resulting particle sizes, while the [Lipid] and [DMSO] affected the colloidal stability. Physicochemical characteristics important for the adjuvant function; the zeta potentials and poly(I:C) adsorption rates were not altered for the formulation samples tested by microfluidic manufacture. Consequently, the CD8^+^ T-cell responses induced against the HPV-16 E7 antigen were not different between CAF09b prepared by high shear mixing and microfluidics.

## Figures and Tables

**Table 1:** A design of experiments (DoE) with CAF09b was designed with the parameters concentration of EtOH [EtOH], total lipid concentration after microfluidics [Lipid] and DMSO concentration [DMSO]. FRR: Flow rate ratio.

Parameter	Abbreviation	Low Level	Middle Level	High Level
EtOH conc. % *v/v* (corresponding FRR)	[EtOH]	20 (4:1)	30 (2.35:1)	40 (1.5:1)
Total lipid conc. after microfluidics *, mg/mL	[Lipid]	6	9	12
DMSO conc. % *v/v*	[DMSO]	0	5	10

* The concentration of lipids (DDA and MMG) in the formulation after assembly on microfluidics but prior to dilution. However, the dilution was immediate in the collection vial and resulted in a final lipid concentration of 2.5/0.5/0.125 mg/mL DDA/MMG/poly(I:C).

**Table 2:** The parameter settings employed in the DoE and the results used for response surface methodology (RSM) analysis. The concentrations of the parameters; lipid, DMSO and EtOH, were measured after formulation on the microfluidic chip but prior to dilution in the collection vial. The formulations were directly diluted to a final concentration of 2.5/0.5/0.125 mg/mL DDA/MMG/poly(I:C), reducing the EtOH concentration accordingly while maintaining the DMSO concentration constant. The samples were analyzed on the day of manufacture, Day 0, for particle size, polydispersity index (PDI) and zeta potential (Zp). On Day 1 and Day 21, the particle size was measured for samples stored at 4 °C and RT, and the size ratio was calculated in relation to the particle size measured on Day 0. On Day 7, the concentration of poly(I:C) was measured on samples stored at 4 °C.

**Sample No.**	**DoE Parameters**			**Day 0**			**Day 1 4 °C**			**Day 21 4 °C**			**Day 1 RT**			**Day 21 RT**			**Day 7 4 °C**
	**[Lipid] mg/mL**	**[DMSO] % *v/v***	**[EtOH] % *v/v***	**Size, nm**	**PDI**	**Zp, mV**	**Size, nm**	**PDI**	**Size Ratio**	**Size, nm**	**PDI**	**Size Ratio**	**Size, nm**	**PDI**	**Size Ratio**	**Size, nm**	**PDI**	**Size Ratio**	**Poly(I:C), mg/mL**
**1**	6	0	40	186	0.34	66	185	0.38	1.0	7082	0.87	38.1	187	0.38	1.0	6350	0.93	34.2	79
**2**	6	5	40	322	0.48	82	521	0.55	1.6	3319	1.00	10.3	313	0.87	1.0	1121	0.94	3.5	87
**3**	6	10	40	158	0.26	57	155	0.26	1.0	474	0.51	3.0	159	0.28	1.0	742	0.78	4.7	69
**4**	9	0	40	181	0.36	61	171	0.44	0.9	175	0.42	1.0	176	0.41	1.0	173	0.41	1.0	131
**5**	9	5	40	174	0.33	72	174	0.31	1.0	200	0.38	1.2	181	0.39	1.0	276	0.61	1.6	148
**6**	9	10	40	198	0.35	59	183	0.36	0.9	961	0.78	4.9	191	0.40	1.0	505	0.52	2.6	126
**7**	12	0	40	183	0.40	68	186	0.33	1.0	204	0.37	1.1	184	0.42	1.0	220	0.41	1.2	119
**8**	12	5	40	194	0.36	58	202	0.32	1.0	209	0.36	1.1	206	0.33	1.1	234	0.39	1.2	105
**9**	12	10	40	138	0.20	58	150	0.21	1.1	144	0.22	1.0	154	0.24	1.1	185	0.31	1.3	103
**10**	6	0	30	59	0.25	64	55	0.22	0.9	61	0.22	1.0	58	0.23	1.0	71	0.21	1.2	124
**11**	6	5	30	52	0.17	45	53	0.18	1.0	55	0.16	1.1	56	0.19	1.1	77	0.34	1.5	101
**12**	6	10	30	83	0.13	54	84	0.15	1.0	90	0.17	1.1	85	0.15	1.0	94	0.21	1.1	67
**13**	9	0	30	82	0.24	62	103	0.26	1.3	150	0.23	1.8	59	0.24	0.7	68	0.24	0.8	107
**14**	9	5	30	58	0.24	65	67	0.22	1.2	100	0.22	1.7	59	0.23	1.0	68	0.25	1.2	110
**15**	9	5	30	56	0.23	45	76	0.23	1.4	112	0.22	2.0	64	0.27	1.2	71	0.27	1.3	75
**16**	9	5	30	65	0.22	56	69	0.21	1.1	98	0.20	1.5	66	0.23	1.0	74	0.25	1.1	108
**17**	9	5	30	49	0.19	45	75	0.22	1.5	116	0.22	2.3	58	0.23	1.2	68	0.25	1.4	81
**18**	9	5	30	62	0.23	55	64	0.19	1.0	96	0.21	1.5	61	0.20	1.0	71	0.23	1.1	86
**19**	9	5	30	52	0.22	60	67	0.22	1.3	102	0.20	1.9	55	0.22	1.0	65	0.23	1.2	110
**20**	9	10	30	52	0.27	52	53	0.28	1.0	60	0.34	1.2	54	0.25	1.0	64	0.21	1.2	109
**21**	12	0	30	57	0.25	50	130	0.28	2.3	235	0.37	4.1	55	0.28	1.0	64	0.28	1.1	121
**22**	12	5	30	71	0.28	57	94	0.22	1.3	144	0.23	2.0	72	0.24	1.0	79	0.26	1.1	140
**23**	12	10	30	56	0.17	52	69	0.19	1.2	99	0.17	1.8	61	0.17	1.1	67	0.16	1.2	142
**24**	6	0	20	42	0.25	59	121	0.24	2.8	3400	1.00	80.3	48	0.26	1.1	75	0.33	1.8	118
**25**	6	5	20	47	0.43	55	79	0.28	1.7	114	0.24	2.4	61	0.35	1.3	66	0.50	1.4	133
**26**	6	10	20	40	0.29	49	45	0.25	1.1	62	0.25	1.5	45	0.25	1.1	64	0.38	1.6	129
**27**	9	0	20	60	0.36	58	355	0.61	5.9	3157	1.00	52.3	45	0.32	0.8	87	0.35	1.4	124
**28**	9	5	20	55	0.28	53	130	0.27	2.4	244	0.34	4.4	47	0.36	0.9	72	0.33	1.3	122
**29**	9	10	20	44	0.36	45	108	0.27	2.5	929	0.81	21.1	52	0.31	1.2	81	0.49	1.8	124
**30**	12	0	20	49	0.27	53	1123	0.84	23.0	2887	1.00	59.3	58	0.32	1.2	117	0.33	2.4	143
**31**	12	5	20	43	0.30	52	124	0.27	2.9	586	0.65	13.7	44	0.27	1.0	52	0.27	1.2	134
**32**	12	10	20	48	0.27	53	97	0.28	2.0	175	0.28	3.7	48	0.27	1.0	56	0.30	1.2	153

## Supplementary Materials

The following are available online at https://www.mdpi.com/1999-4923/12/12/1237/s1, Figure S1: RSM model of samples manufactured at 30% EtOH at day 7 of storage at 4°C. Figure S2: Release of poly(I:C) from CAF09b after SDS treatment. Table S1: Statistical significance of parameters in the RSM models.
