# Supplementary material for: Applying Microfluidics for the Production of the Cationic Liposome-Based Vaccine Adjuvant CAF09b
Source: Pharmaceutics. 2020 Dec 19;12(12):1237. doi: 10.3390/pharmaceutics12121237 (PMC7767004; doi:10.3390/pharmaceutics12121237)
Supplement: Supplementary file 1 [file pharmaceutics-12-01237-s001.pdf]

pharmaceutics

MDPI

Supplementary Materials: Applying Microfluidics for the Production of the Cationic Liposome-Based Vaccine Adjuvant CAF09b

Signe Tandrup Schmidt, Dennis Christensen and Yvonne Perrie

(a)

Sample no.	DoE parameters			Day 7, 4°C			Sample no.	DoE parameters			Day 7, 4°C		
	[Lipid] mg/ml	[DMSO] % v/v	[Lipid] mg/ml	Size, nm	PDI	Size ratio		[Lipid] mg/ml	[DMSO] % v/v	[Lipid] mg/ml	Size, nm	PDI	Size ratio
1	6	0	40	987	0.72	5.3	17	9	5	30	101	0.20	2.0
2	6	5	40	1673	0.93	5.2	18	9	5	30	79	0.22	1.3
3	6	10	40	400	0.40	2.5	19	9	5	30	89	0.22	1.7
4	9	0	40	174	0.41	1.0	20	9	10	30	58	0.29	1.1
5	9	5	40	176	0.31	1.0	21	12	0	30	176	0.26	3.1
6	9	10	40	228	0.37	1.2	22	12	5	30	121	0.23	1.7
7	12	0	40	188	0.33	1.0	23	12	10	30	83	0.19	1.5
8	12	5	40	197	0.34	1.0	24	6	0	20	2234	0.93	52.8
9	12	10	40	137	0.20	1.0	25	6	5	20	100	0.26	2.1
10	6	0	30	57	0.21	1.0	26	6	10	20	55	0.25	1.4
11	6	5	30	53	0.16	1.0	27	9	0	20	1670	1.00	27.6
12	6	10	30	86	0.14	1.0	28	9	5	20	209	0.29	3.8
13	9	0	30	132	0.23	1.6	29	9	10	20	129	0.26	2.9
14	9	5	30	87	0.23	1.5	30	12	0	20	3744	0.93	76.9
15	9	5	30	93	0.22	1.7	31	12	5	20	372	0.57	8.7
16	9	5	30	84	0.20	1.3	32	12	10	20	144	0.27	3.0

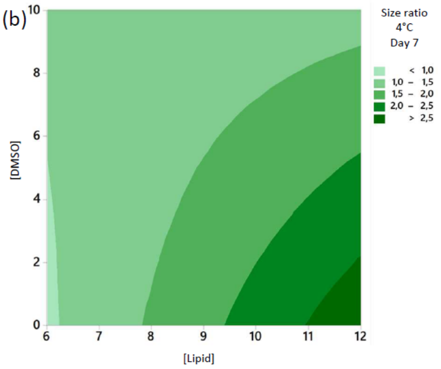

Figure S1. (**a**) Size, PDI and size ratio for all samples on day 7 at 4°C storage. (**b**) RSM model of the size ratio of samples on day 7, manufactured at 30% EtOH and stored at 4°C.

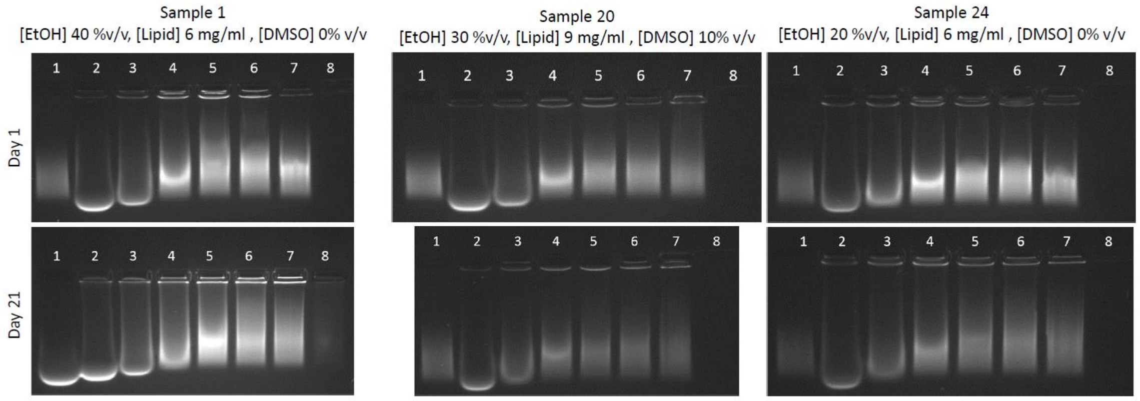

Figure S2. Release of poly(I:C) from CAF09b after SDS treatment. The samples were treated with 0, 0.1, 0.25, 0.5, 1, 2, and 3% SDS, and analyzed with agarose (1%) gel electrophoresis the day after preparation and 21 days after preparation, storage at 4°C. Lane 1: Poly(I:C) control, lane 2–8: CAF09b treated with 3, 2, 1, 0.5, 0.25, 0.1 and 0% SDS, respectively.

Table S1. Statistical significance of parameters in the RSM models.

**Figure 1a**	
Source	p-value
Model	0.000
Linear	0.000
[EtOH]	0.000
[DMSO]	0.001
Square	0.000
[EtOH]*[EtOH]	0.000
2-way interaction	0.007
[EtOH]*[DMSO]	0.007
Error	
Lack-of-fit	0.177
**Figure 1b**	
Source	p-value
Model	0.001
Linear	0.074
[EtOH]	0.123
[Lipid]	0.353
[DMSO]	0.041
Square	0.001
[EtOH]*[EtOH]	0.001
2-way interaction	0.004
[EtOH]*[DMSO]	0.003
[Lipid]*[DMSO]	0.084
Error	
Lack-of-fit	0.044
**Figure 1c**	
Source	p-value
Model	0.004
Linear	0.004
[EtOH]	0.004
[DMSO]	0.046
Square	0.082
[EtOH]*[EtOH]	0.082
Error	
Lack-of-fit	0.962
**Figure 2a**	
Source	p-value
Model	0.017
Linear	0.007
[Lipid]	0.006
[DMSO]	0.041
Square	0.729
[Lipid]*[Lipid]	0.574
[DMSO]*[DMSO]	0.797
2-way interaction	0.024
[Lipid]*[DMSO]	0.024
Error	
Lack-of-fit	0.320

Figure 2b	
Source	p-value
Model	0.006
Linear	0.002
[Lipid]	0.002
[DMSO]	0.018
Square	0.925
[Lipid]*[Lipid]	0.760
[DMSO]*[DMSO]	0.934
2-way interaction 0.019	0.019
[Lipid]*[DMSO]	0.019
Error	
Lack-of-fit	0.145
**Figure 2c**	
Source	p-value
Model	0.243
Linear	0.200
[Lipid]	0.867
[DMSO]	0.083
Square	0.179
[Lipid]*[Lipid]	0.373
[DMSO]*[DMSO]	0.072
2-way interaction	0.690
[Lipid]*[DMSO]	0.690
Error	
Lack-of-fit	0.179
**Figure 2d**	
Source	p-value
Model	0.262
Linear	0.269
[Lipid]	0.244
[DMSO]	0.251
Square	0.159
[Lipid]*[Lipid]	0.253
[DMSO]*[DMSO]	0.066
2-way interaction	0.622
[Lipid]*[DMSO]	0.622
Error	
Lack-of-fit	0.081
**Figure 3a**	
Source	p-value
Model	0.003
Linear	0.003
[EtOH]	0.013
[Lipid]	0.007
Error	
Lack-of-fit	0.082
**Supplementary figure 1b**	
Source	p-value
Model	0.010

Linear	0.003
[Lipid]	0.003
[DMSO]	0.027
Square	0.989
[Lipid]*[Lipid]	0.946
[DMSO]*[DMSO]	0.929
2-way interaction	0.025
[Lipid]*[DMSO]	0.025
Error	
Lack-of-fit	0.376
